# Supplementary material for: Flow of cerebrospinal fluid is driven by arterial pulsations and is reduced in hypertension
Source: Nat Commun. 2018 Nov 19;9:4878. doi: 10.1038/s41467-018-07318-3 (PMC6242982; doi:10.1038/s41467-018-07318-3)
Supplement: Supplementary file 1 — Supplementary Information [file 41467_2018_7318_MOESM1_ESM.pdf]

Supplementary Information for “Flow of cerebrospinal fluid is driven by arterial pulsations and is reduced in hypertension” by Mestre *et al.*

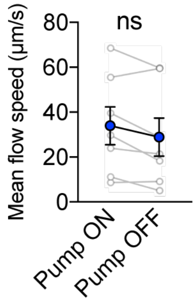

Supplementary Figure 1. Microsphere infusion does not affect perivascular CSF flow speed. CSF flow measurements collected during microsphere infusion into the cisterna magna (Pump ON) and after the pump was turned off (Pump OFF). Paired t-test, *P* = 0.0624, ns: not significant, mean ± SEM, n = 7 mice. All velocities plotted or discussed elsewhere were measured after the pump was turned off.

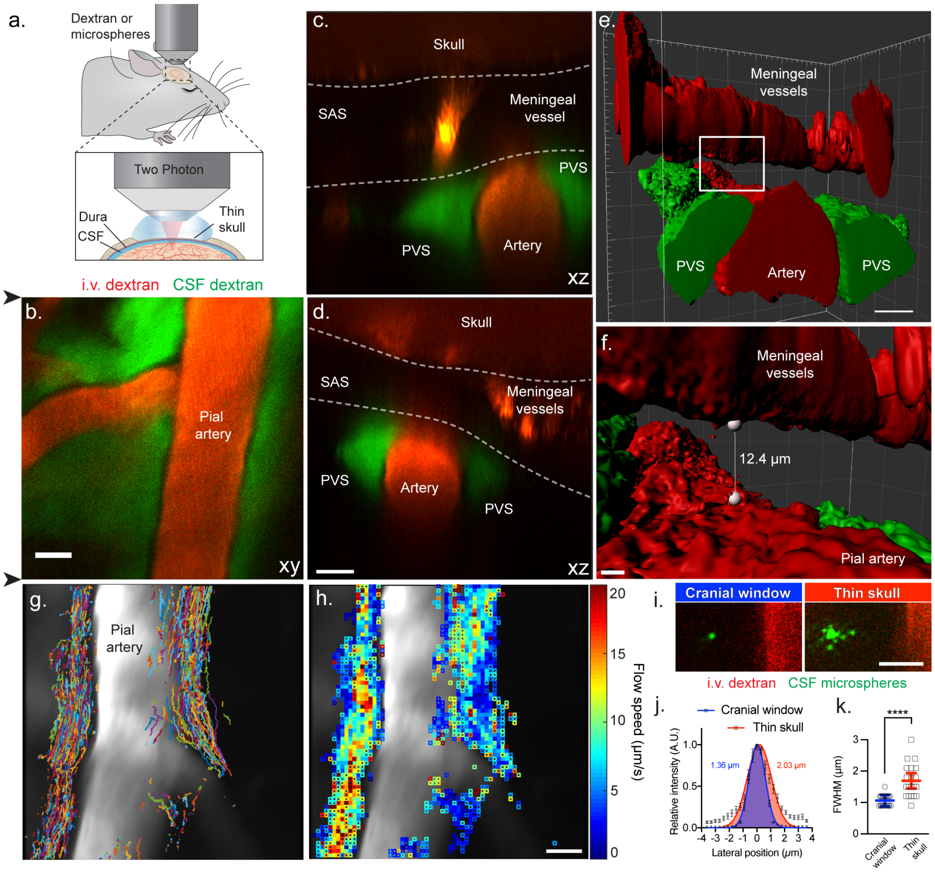

Supplementary Figure 2. PVS morphology and flow are unchanged in thinned skull preparations but show a reduced radial point spread function compared to cranial windows. (a) Anesthetized mice were imaged through a thinned skull after receiving an intracisternal injection of either 2,000 kDa dextran or fluorescent microspheres and iv dextran to label the vasculature**. (b)** CSF tracers distribute along PVSs of pial arteries with a similar morphology to cranial window experiments**.** Scale bar: 20 µm.**(c)** Orthogonal projections of a proximal (top black arrow) and **(d)**distal (bottom black arrow) location of same data shown in (b) indicate subarachnoid space (SAS) between the skull and the pial surface and two subpial PVSs at each side of the artery. Scale bar: 20 µm.**(e)** Three-dimensional reconstruction of (b) and **(f)**enlargement of inset in (e) show a gap between the dural vasculature and the pial artery suggesting that the two PVSs are not an artifact from the cranial window preparation**. (g)** Particle tracking velocimetry reveals PVS widths and **(h)**bulk flow speeds comparable to measurements using cranial windows. Scale bar: 20 µm.**(i)** Representative images of single particles from a cranial window (left) and thin skull (right) experiment showing greater optical aberrations in the latter. Scale bar: 10 µm.**(j)**Average relative intensity distributions of 20 random 1 µm microspheres**,** mean ± SEM. Gaussian functions fit to mean data for a cranial window (R^2= 0.91) and thin skull (R^2= 0.69) experiment. Full width at half maximum (FWHM) for the fitted function is presented on the graph and color-coded to the experiment type. **(k)** FWHM size of microspheres in cranial window (1.065 ±0.04 µm) and thin skull (1.695 ±0.12 µm) experiments**.** Unpaired t-test, *****P*< 0.0001, mean ± SEM,n = 20 microspheres from a cranial window or a thin skull experiment.

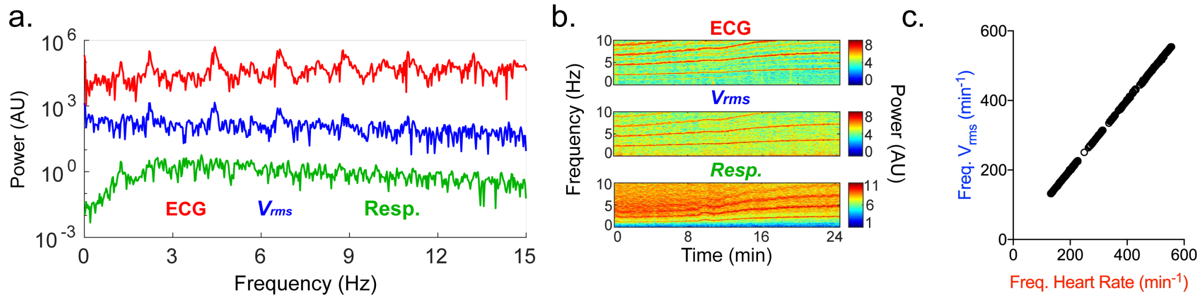

Supplementary Figure 3. CSF flow pulses in synchrony with the cardiac cycle. (a) Power spectra in arbitrary units (AU) computed for a 1-minute sample of the ECG, v_{rms}, and respiration. The spectra for the ECG and v_{rms} are remarkably similar in both their primary frequency ($f = 2.2$ Hz) and their harmonics ($f = 4.4, 6.6, \ldots$ Hz). **(b)** Spectrograms from the full 25-minute time series for each of the 3 signals. As the heart rate increases from 2.2 to 3.6 Hz (top plot, bottom horizontal red line), the frequency of v_{rms} and respiration rate also increase. **(c)** Dominant frequencies of the ECG and v_{rms} for several 1-minute intervals from n=8 mice, indicating that the frequency of oscillations in v_{rms} is locked to the heart rate. Pearson's correlation coefficient, R^2 = 0.999, P< 0.0001.

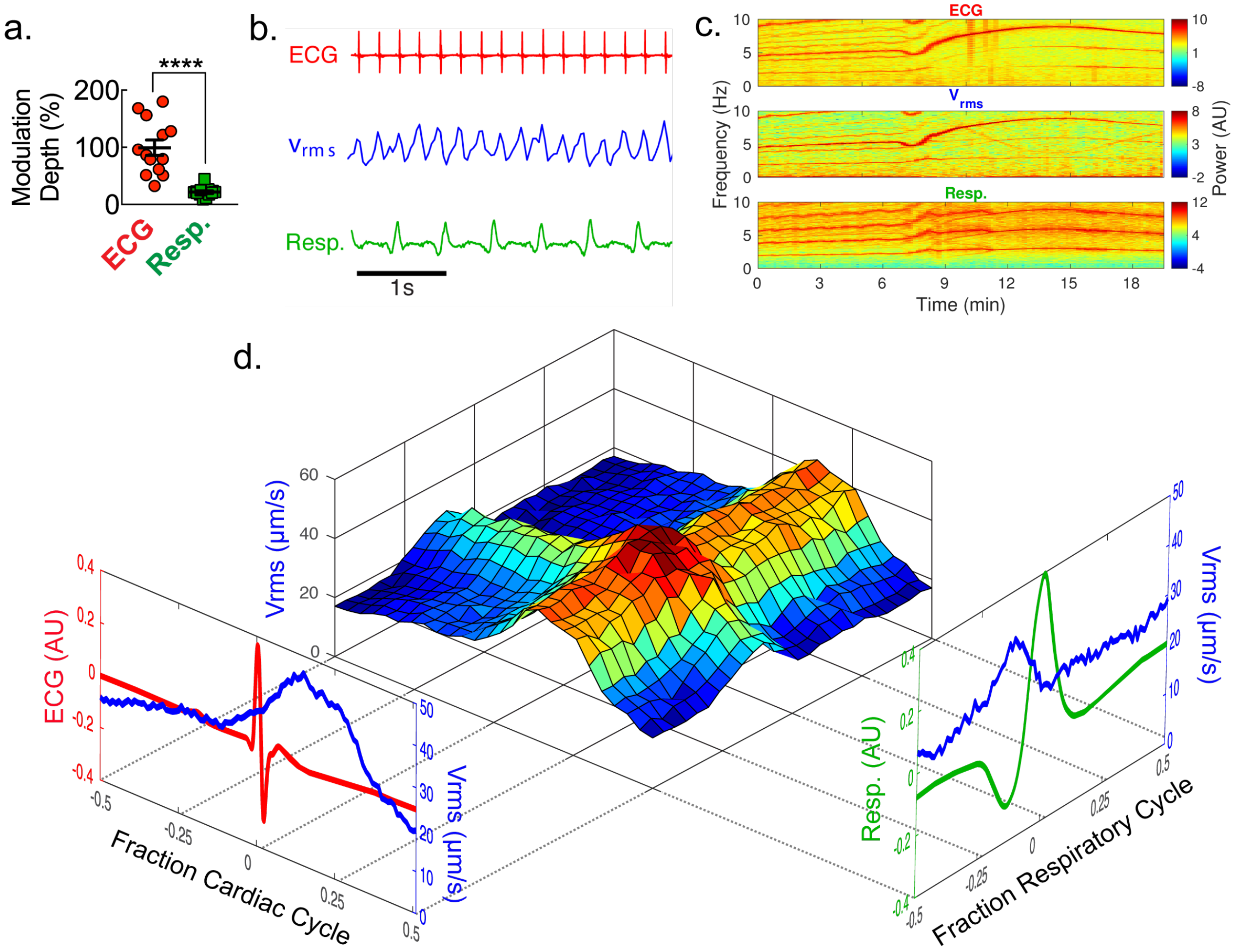

Supplementary Figure 4. Respiratory modulation of CSF fluid flow. (a) Modulation depth of v_{rms} over the cardiac and respiratory cycles, calculated as (max-min)/mean. Unpaired t-test, *****P* < 0.0001, mean ± SEM, n = 13 mice. **(b)** Representative traces of ECG (red curve), v_{rms} (blue curve), and respiration (green curve) for the mouse with the largest respiratory modulation observed. **(c)** Spectrograms from the full 20-minute time series for each of the signals. A weak red line corresponding to the primary respiration frequency ($f = 2$ Hz) is visible in the top two plots, indicating that the respiratory cycle affects the heart rate and v_{rms}. **(d)** v_{rms} conditionally averaged over the cardiac and respiratory cycles. The protruding plots show v_{rms} averaged over the cardiac (left) and respiratory (right) cycles alone, which show 86% and 45% modulation, respectively.

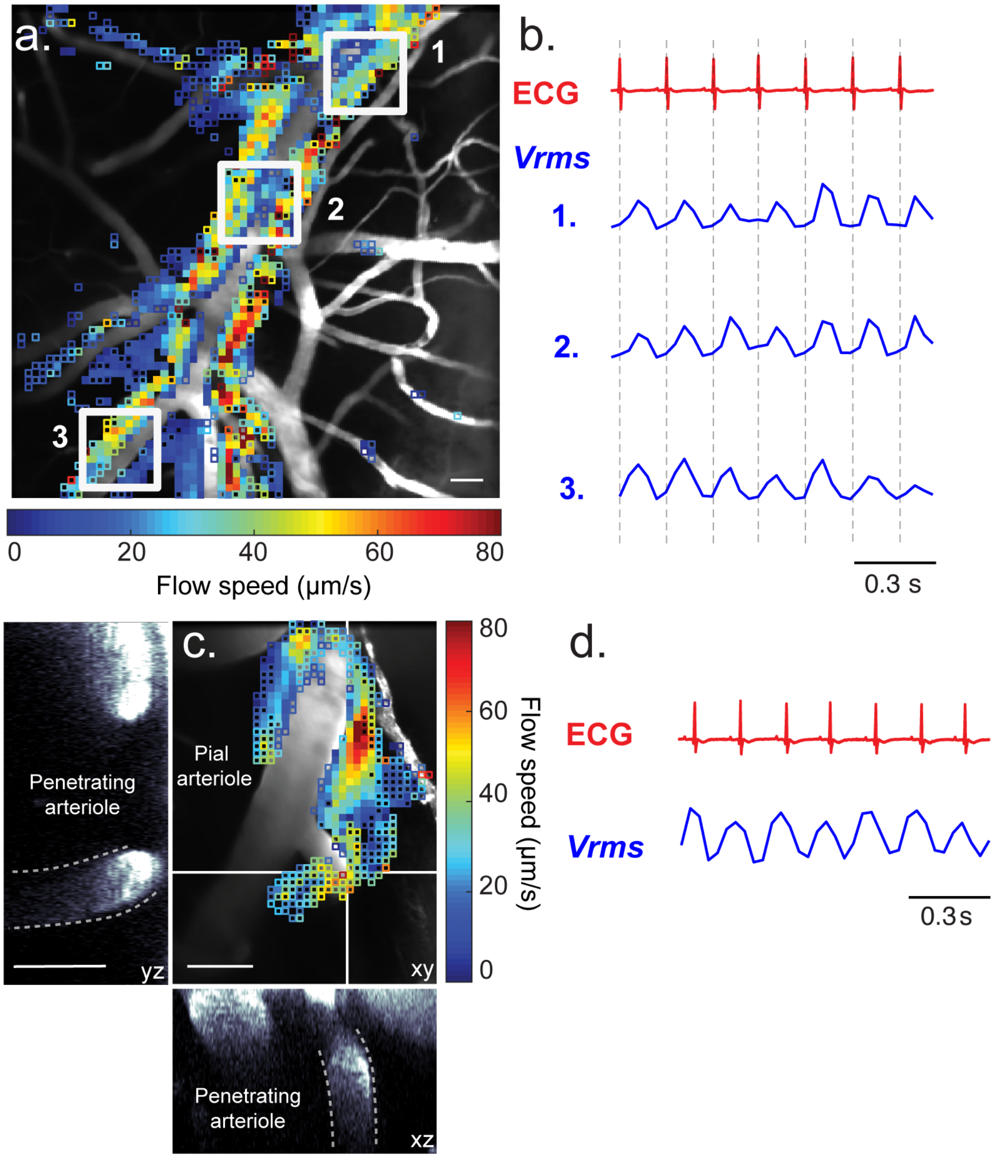

Supplementary Figure 5. Bulk flow continues down smaller pial arteries towards penetrating arterioles. (a) The local time-averaged flow speed in PVSs around a smaller pial artery, both proximal and distal to bifurcations, is similar to the speed in PVSs around the MCA (Fig. 1d). Scale bar: 40 µm. **(b)** The root-mean-square velocity (v_{rms}) in the three regions indicated in (a), which are separated by several bifurcations, pulses in synchrony with the R wave of the electrocardiogram (ECG), like flow near the MCA (Fig. 3). **(c)** The local time-averaged flow speed in PVSs around a pial arteriole as it dives down into the parenchyma is also similar to the speed near the MCA. Scale bar: 40 µm. **Orthogonal projections** of a z-stack acquired after imaging of (c). Scale bar: 50 µm. **(d)** The v_{rms} where the penetrating arteriole descends into cortex also pulses in synchrony with the heart. Microspheres (1 µm) aggregate at the entry site of the penetrating PVS, preventing CSF flow measurements at deeper locations.

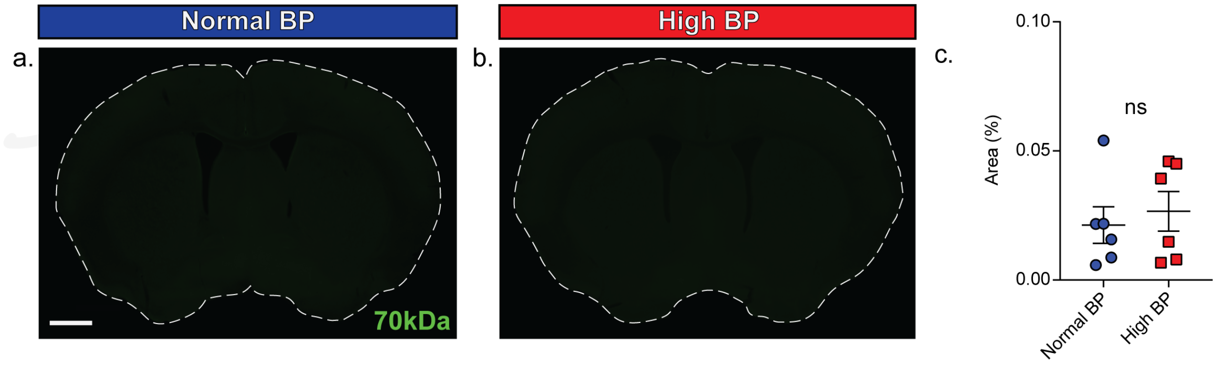

Supplementary Figure 6. Acute hypertension does not disrupt the blood-brain barrier (BBB). Coronal sections from mice that received either a 30-minute intravenous infusion of **(a)** PBS or **(b)** Ang-II following an i.v. injection of 1% FITC-Dextran (70kDa). Both images are nearly black because almost no dextran crossed the BBB. Scale bar: 1 mm. **(c)** Coronal sections from each group were quantified by measuring the percent area using an automated thresholding method, mean ± SEM, $P>0.05$; Unpaired t-test, ns: not significant. n=6/group.

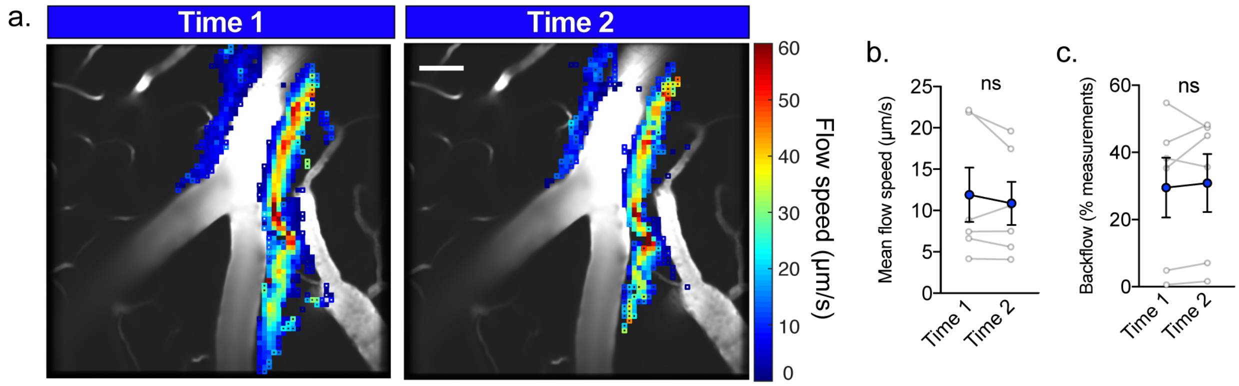

Supplementary Figure 7. Control mice show no significant change in mean flow speed over time. (a) Local time-averaged flow speeds for two intervals of normal blood pressure in a control mouse. Closed squares indicate regions with at least 20 measurements; open squares, fewer than 20. Scale bar: 40 µm. **(b)** The mean flow speed does not change significantly from one interval to the next. Paired t-test, *P* = 0.3022, ns: not significant, mean ± SEM, n=6. **(c)** Measurements of percent of backflow for each time interval show no significant change. Paired t-test, *P* = 0.5964, ns: not significant, mean ± SEM, n=6.

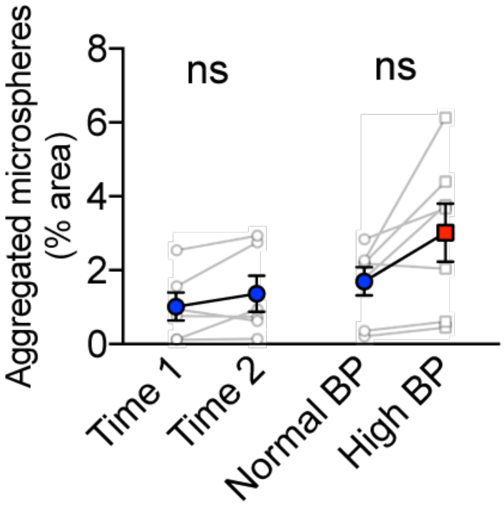

Supplementary Figure 8. Microsphere aggregation in the PVS occurs over time and is similar in both hypertension and control groups. Efforts were made to prevent microspheres from aggregating, but a small number become adhered to the PVS wall and remained stationary. We were careful to remove measurements of stagnant particles by masking such regions and also removing particle tracks with small displacements (see Methods). Aggregated microspheres were quantified as % of covered area using a thresholding approach. In mice that received a control infusion of saline (left) and in mice that received an infusion of Ang-II (right) there was an equal degree of microsphere aggregation during the experimental time period. Two-way repeated measures ANOVA, interaction term, ns: not significant, P = 0.149, mean ± SEM, n= 6-7.

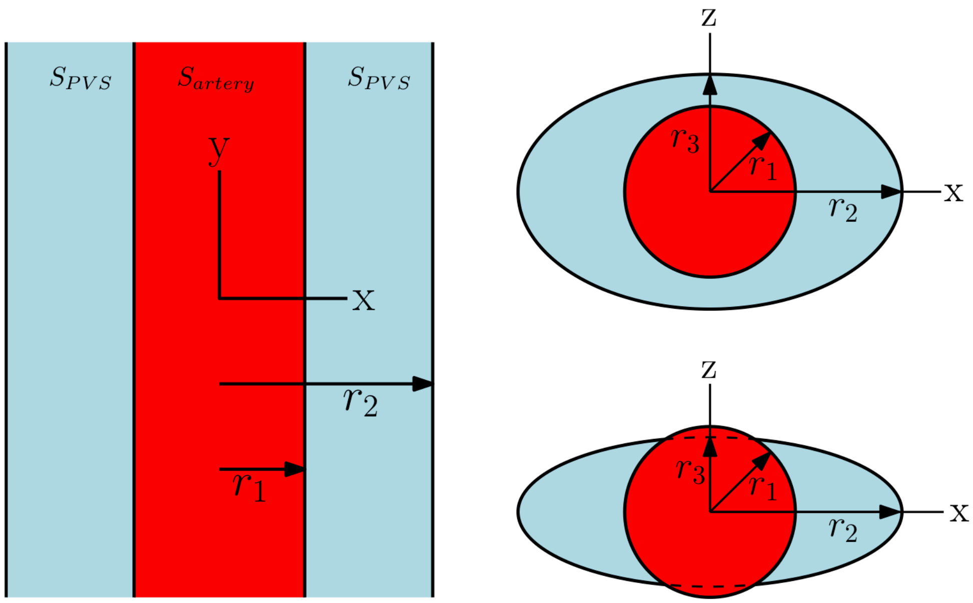

Supplementary Figure 9. A simplified geometric model of the PVS around the MCA. Sketches of lateral (left) and cross-sectional (right) areas, comparable to Fig. 1c and 1d, respectively. The size of the PVS is parameterized by the radius of the artery (r_1) and the semi-major (r_2) and semi-minor (r_3) axes of an ellipse forming the outer boundary of the PVS. Prior modeling studies have assumed $r_3 = r_2$, such that the PVS is a circular annulus. Our results suggest $r_2 > r_3 \approx r_1$ and in some cases $r_3 < r_1$ (bottom right sketch).

Mouse	Particle Track Total	Measurement Count Total
Control Mouse 1	1671	51667
Control Mouse 2	41770	733183
Control Mouse 3	2783	122245
Control Mouse 4	3122	95093
Control Mouse 5	1538	40591
Control Mouse 6	25400	393518
Hypertension Mouse 1	23920	489159
Hypertension Mouse 2	44107	1150323
Hypertension Mouse 3	8770	234809
Hypertension Mouse 4	36995	328868
Hypertension Mouse 5	32878	384797
Hypertension Mouse 6	18193	400086
Hypertension Mouse 7	15316	190830
Mean ± SEM	19728 ± 4358	355013 ± 85957

Supplementary Table 1. Particle tracking velocimetry measurements. The total number of particle tracks is roughly equal to the number of microspheres that enter the PVS during imaging; the count may differ due to the PTV algorithm failing to track some microspheres or identifying the same microsphere as two different ones at different moments. The measurement count total is the total number of microsphere position and velocity measurements across all tracks for the entire imaging time. Stagnant microspheres have been excluded from the statistics. Microspheres appeared almost exclusively in the PVS of the MCA and its downstream branches with only about 2.5 ± 1.6% of particles appearing outside the space.

Supplementary Discussion.

Volume of the Perivascular Space

One important result of our experiments is that the size of the PVS, as measured by microsphere trajectories (Fig. 1b) or injected dye (Fig. 2a), is much larger than has been previously reported. Here we describe an idealized geometric model of the PVS which allows us to relate the *in vivo* lateral area ratios presented in Fig. 2k to an approximate volume ratio. Consider the idealized geometry shown in Supplementary Fig. 9. The circle represents the outer boundary of the artery and the ellipse represents the outer wall of the PVS; each of these extends uniformly in the *y*-direction. Let r_1 denote the radius of the circle and r_2 and r_3 denote the semi-major and semi-minor axes of the ellipse, respectively. Then the cross-sectional area of the artery is $A_{artery} = \pi r_1^2$ and the cross-sectional area of the PVS is $A_{PVS} = A_{ellipse} - A_{artery} = \pi(r_2 r_3 - r_1^2)$. Hence, the ratio of the cross-sectional areas of the artery and the PVS, which is also the ratio of their respective volumes (per unit length), is

$$\frac{V_{PVS}}{V_{artery}} = \frac{A_{PVS}}{A_{artery}} = \frac{r_2 r_3}{r_1^2} - 1. \quad (1)$$

Note that if we take $r_2 = r_3 > r_1$ we have concentric circular cylinders, and if we take $r_3 = r_1 < r_2$ the circle is tangent to the ellipse at the top and bottom, i.e. the PVS vanishes at the top and bottom (we refer to this as *two-lobe* geometry). The ratio of the lateral areas (in the x-y plane) of the PVS and the artery is

$$\alpha = \frac{S_{PVS}}{S_{artery}} = \frac{2(r_2 - r_1)L}{2 r_1 L} = \frac{r_2}{r_1} - 1. \quad (2)$$

From our experimental data, we find approximately $\alpha = 1.4$, which gives $r_2/r_1 = 2.4$. Using this value, we can compute the volume ratio for two cases.

For the case of concentric cylinders, which has been widely assumed in modeling studies[1-3], we have $r_2 = r_3 = 2.5r_1$, and equation (4) gives the volume ratio

$$\frac{V_{PVS}}{V_{artery}} = \frac{A_{PVS}}{A_{artery}} = (2.4)^2 - 1 = 4.76. \quad (3)$$

For the case of a two-lobe geometry, $r_3 = r_1$ and equation (4) gives

$$\frac{V_{PVS}}{V_{artery}} = \frac{A_{PVS}}{A_{artery}} = 2.4 - 1 = 1.4. \quad (4)$$

Our observation that very few particles cross above the artery suggests the concentric cylinder model is incorrect, at least near the MCA. The PVS appears to be closer to a two-lobe geometry, which is supported by *in vivo* x-z cross sections of dye reconstructed using z-stacks of x-y images (e.g., Fig. 2b). Although in some cases reconstructions suggest that r_3 is slightly smaller than r_1, they are often comparable. We are confident in claiming that the volume (and cross-sectional area) of the PVS is about the same as that of the artery.

Polystyrene Microspheres are Accurate Cerebrospinal Fluid Tracers

The Stokes number is a positive, dimensionless quantity which characterizes how accurately a particle suspended in a fluid flow tracks the motion of the fluid (small values indicate accurate tracers). The Stokes number is defined as

$$St = \frac{2}{9}\frac{\rho_m}{\rho_f}\left(\frac{d}{L}\right)^2 Re \quad (5)$$

where $\rho_m = 1060$ kg m^{-3} is the density of the microspheres, $\rho_f = 993$ kg m^{-3} is the density of the cerebrospinal fluid, $d = 1\times10^{-6}$ m is the diameter of the microspheres, $L =$

4.4×10^{-5} m is the average PVS width (Fig. 2d), and $Re = 0.0012$ is the Reynolds number (Fig. 1k). Hence, the Stokes number is $St = 1.5\times10^{-7}$, which indicates that the microspheres track the motion of the cerebrospinal fluid very well.

Consistency with Prior Modeling Studies

Our finding of net perivascular pumping of CSF in the PVS in the same direction as the blood flow is consistent with theoretical considerations. Computational models of flows in the perivascular space driven solely by arterial pulsations show that, although there are localized negative velocities at certain times, the average flow is in the direction of the pulse wave[1,2,4,5]. Analytical models of perivascular pumping in narrow channels (i.e., narrow compared to the wavelength of the arterial traveling wave, which is the case here) show that in general the net flow consists of an arterial-wall-driven component in the direction of the arterial wave plus additional flow driven by any overall pressure gradient that may be present[6]. Wang and Olbricht[1] present an analytical solution for perivascular pumping in a circular annulus model of the PVS, from which we obtain the following expression for the average flow velocity, driven purely by the arterial wall motion:

$$v = \frac{2R_1^4}{(R_2^2 - R_1^2)^2}\left(\frac{b}{R_1}\right)^2 c, \qquad (6)$$

where R_1 and R_2 are the inner and outer radii of the annulus, b is the half-amplitude of the arterial traveling wave, and c is the speed of the arterial traveling wave. Typical values from our data are $R_1 = 30$ µm, $R_2 = 70$ to 80 µm (average radius of an elliptical PVS), $b = 0.3$ µm, and $c = 1$ m s^{-1}, from which we obtain average flow speeds in the range $v = 5$ to 10 µm s^{-1}, which is remarkable agreement with our experimental measurements,

considering the simplifying assumptions in the model. Note also that the expression above implies that the average flow speed is greater in a narrower annulus, in agreement with our velocity profiles at locations where the width of the PVS is narrower (Fig. 1e). Thus, modeling predictions suggest that perivascular pumping alone may account for the observed flows, although given the uncertainties in the parameter values, we cannot rule out the possibility that the flow is partially driven by an overall pressure gradient due to, say, the production of CSF. We note that two-photon imaging is not fast enough to precisely measure the speed at which the arterial wave propagates through the vasculature. Our estimate of this speed is 1 m/s, which comes from an approximation of the heart-to-brain distance divided by the 35 ms delay observed. This is an important modeling parameter that should be precisely measured in future work.

<u>Previous qualitative studies of cerebrospinal fluid flow in perivascular spaces</u>

Many previous studies have inferred CSF flow in PVSs by evaluating the influx of fluorescent dye[7-13]. In cases where CSF influx is quantified, researchers typically do so using mean pixel intensities or thresholded area/volume measurements. A limitation of such approaches is the inability to measure spatially and temporally resolved flow velocities. Measurement techniques such as particle tracking velocimetry (PTV) or particle image velocimetry (PIV) require seeding a fluid flow with particles that generate contrast when imaged (e.g., bright particles with a dark background). This approach cannot be applied to dyes/tracers which tend to generate smooth intensity profiles with little to no contrast in the flow. However, the use of larger fluorescent particles (e.g.,

diameter 1 μm) can generate the contrast necessary, enabling quantitative flow measurements when combined with high temporal and spatial resolution imaging.

One other study, contemporary with ours, made initial strides towards characterizing CSF flows in PVSs using fluorescent microspheres. The *qualitative* results reported by Bedussi et al.[14] are consistent with the *quantitative* results reported in this study. Specifically, Bedussi et al. tracked an average of six particles per animal (n=5) in PVSs and observe that the particles move in a pulsatile manner at frequencies similar to cardiac cycle frequencies (although imaging was not synchronized to heart rate measurements), with net motion in the same direction as blood flow. Respiration was driven with a ventilator at a constant rate; its effects were not addressed. Because the experiments involved imaging through a thinned skull using single photon microscopy, resolving individual micron-sized microspheres would have been difficult, and the tracked particles could be large agglomerations of microspheres (Supplementary Fig. 2i-k). The two particles shown in their Fig. 1b appear to each have a diameter near 10 μm, which would substantially alter the flow through a space just 40 μm wide (Fig. 2d). In contrast, our study involved tracking 20000 individual microspheres on average (Supplementary Table 1), and we have rigorously shown that the frequency of the flow pulsatility is locked to the cardiac cycle using hundreds of measurements over tens of minutes (Extended Data Fig. 3c). Bedussi et al. report 2 s of measurements, which is an insufficient interval for generating power spectra or spectrograms (Supplementary Fig. 3a-b) to accurately quantify the frequencies of the heart beat or CSF flow. Of particular concern is their report of arterial blood velocities in the range of 0.9 to 5.5 μm/s, which is an underestimate by approximately a factor of 1000[15]. Furthermore, the conclusions of Bedussi et al. directly

contradict ours: this study provides ample evidence demonstrating that CSF is driven primarily by perivascular pumping in the PVSs, which directly refutes their conclusion that in the absence of valves, pulsations cannot create net flow, and that the flow is driven entirely by CSF production in the choroid plexus.

References

1. Wang, P. & Olbricht, W.L. Fluid mechanics in the perivascular space. *J Theor Biol* **274**, 52-57 (2011).
2. Bilston, L.E., Fletcher, D.F., Brodbelt, A.R. & Stoodley, M.A. Arterial pulsation-driven cerebrospinal fluid flow in the perivascular space: a computational model. *Comput Methods Biomech Biomed Engin* **6**, 235-241 (2003).
3. Jin, B.J., Smith, A.J. & Verkman, A.S. Spatial model of convective solute transport in brain extracellular space does not support a "glymphatic" mechanism. *J Gen Physiol* **148**, 489-501 (2016).
4. Schley, D., Carare-Nnadi, R., Please, C.P., Perry, V.H. & Weller, R.O. Mechanisms to explain the reverse perivascular transport of solutes out of the brain. *J Theor Biol* **238**, 962-974 (2006).
5. Asgari, M., de Zelicourt, D. & Kurtcuoglu, V. Glymphatic solute transport does not require bulk flow. *Sci Rep* **6**, 38635 (2016).
6. Jaffrin, M.Y. & Shapiro, A.H. Peristaltic Pumping. *Annu Rev Fluid Mech* **3**, 13-& (1971).
7. Iliff, J.J.*, et al.* A paravascular pathway facilitates CSF flow through the brain parenchyma and the clearance of interstitial solutes, including amyloid beta. *Sci Transl Med* **4**, 147ra111 (2012).
8. Ichimura, T., Fraser, P.A. & Cserr, H.F. Distribution of extracellular tracers in perivascular spaces of the rat brain. *Brain Res* **545**, 103-113 (1991).
9. Hadaczek, P.*, et al.* The "perivascular pump" driven by arterial pulsation is a powerful mechanism for the distribution of therapeutic molecules within the brain. *Mol Ther* **14**, 69-78 (2006).
10. Iliff, J.J.*, et al.* Cerebral arterial pulsation drives paravascular CSF-interstitial fluid exchange in the murine brain. *J Neurosci* **33**, 18190-18199 (2013).
11. Bedussi, B.*, et al.* Paravascular channels, cisterns, and the subarachnoid space in the rat brain: A single compartment with preferential pathways. *J Cereb Blood Flow Metab* **37**, 1374-1385 (2017).
12. Rennels, M.L., Gregory, T.F., Blaumanis, O.R., Fujimoto, K. & Grady, P.A. Evidence for a 'paravascular' fluid circulation in the mammalian central nervous system, provided by the rapid distribution of tracer protein throughout the brain from the subarachnoid space. *Brain Res* **326**, 47-63 (1985).
13. Arbel-Ornath, M.*, et al.* Interstitial fluid drainage is impaired in ischemic stroke and Alzheimer's disease mouse models. *Acta Neuropathol* **126**, 353-364 (2013).

14. Bedussi, B., Almasian, M., de Vos, J., VanBavel, E. & Bakker, E.N. Paravascular spaces at the brain surface: Low resistance pathways for cerebrospinal fluid flow. *J Cereb Blood Flow Metab*, 271678X17737984 (2017).
15. Santisakultarm, T.P.*, et al.* In vivo two-photon excited fluorescence microscopy reveals cardiac- and respiration-dependent pulsatile blood flow in cortical blood vessels in mice. *Am J Physiol Heart Circ Physiol* **302**, H1367-1377 (2012).
