# Supplementary material for: Flow of cerebrospinal fluid is driven by arterial pulsations and is reduced in hypertension
Source: Nat Commun. 2018 Nov 19;9:4878. doi: 10.1038/s41467-018-07318-3 (PMC6242982; doi:10.1038/s41467-018-07318-3)
Supplement: Supplementary file 3 — Description of Additional Supplementary Files [file 41467_2018_7318_MOESM3_ESM.pdf]

Description of Additional Supplementary Files

File Name: Supplementary Movie 1
Description: **PVS structure and flow during perfusion-fixation.** An anesthetized mouse received intracisternal fluorescent Texas Red-dextran (2,000 kDa; green) and vascular labeling with FITC-dextran (red). Two-photon imaging of the tracer showed pulsatile, forward flow *in vivo*. The absence of fluorescence between the CSF tracer and the intravascular dextran is due to the tracer being outside the artery wall. Next, phosphate-buffered saline (PBS) was infused through an arterial catheter. PBS perfusion shows the removal of the FITC-dextran and backflow of the tracer. Upon changing to PFA, the drop in intraluminal pressure causes the vessel to collapse and the tracer to rapidly redistribute around the artery wall. PFA perfusion causes the perivascular space to shrink and flow continues.

File Name: Supplementary Movie 2
Description: **Simultaneous recording of CSF flow with ECG and respiration.** Two-photon microscopy imaging (left) and synchronized physiological measurements (right). Trajectories from particle tracking velocimetry are indicated by colored curves tracking the microspheres. Measured root-mean-square velocity v_{rms} peaks occur soon after every R wave of the ECG. Respiration peaks are not highly correlated with v_{rms} peaks.

File Name: Supplementary Movie 3
Description: **CSF flow before and after acute arterial hypertension.** Two-photon microscopy imaging (left) and synchronized physiological measurements (right) for normal (top) and high (bottom) blood pressure. Trajectories from particle tracking velocimetry are indicated by colored curves tracking the microspheres. An increase in backflow (i.e., instantaneous reverse flow) in the case of hypertension is apparent by comparing microsphere motions in the top and bottom frames

File Name: Supplementary Movie 4
Description: **Perivascular pumping animation.** Cerebrospinal fluid bulk flow is driven by perivascular pumping in the PVSs of pial arteries. The PVS is lined by astrocyte endfeet expressing aquaporin-4 (AQP-4) water channel. (**Inset**) A portion of the pial artery is magnified, showing fluorescent particles being transported through the PVS. Electrocardiogram (ECG) and artery diameter measurements show the dilation of the arterial wall about 35 ms after ventricular contraction. Microsphere velocities are shown as root-mean-square averages (v_{rms}).
